# Appendicitis—Its Diagnosis, Pathology and Treatment

**Published:** 1892-09

**Authors:** 


### APPENDICITIS—ITS DIAGNOSIS, PATHOLOGY, AND TREATMENT.

---

(*New Albany Medical Herald*, May, 1892, p. 435.) Dr. L. S. McMurtry gives a brief *resume* of this subject. Appendicitis, in the writer's opinion, is the most frequent of all the causes of peritonitis in the male and a frequent cause of the same in the female. This inflammation may vary from the simple catarrhal form with infiltration of the sub-mucous tissues to perforation and complete gangrene of the part. The course of the case may be acute, sub-acute or chronic, giving a clinical history which may be characterized as fulminant, explosive, recurrent, or mild. In diagnosticating appendicitis, it is impossible to give an accurate estimate of the extent and severity of the lesions. Sometimes the condition may be mistaken for simple intestinal disturbance and passed by unnoticed. On the other hand, cases which may be quite severe and characterized by pain, tenderness, swelling, and febrile action may yet terminate in resolution and complete recovery. In the latter class, inflammation is very apt to recur. Whether it is best to operate, should be determined more by the grade of inflammation than by the time it has existed. "When a diagnosis has been made and three days have elapsed without diminished pulse-rate and temperature, operation should be done." The writer believes that operation involves less danger than delay, and should be resorted to in all cases in which a high grade of inflammation is persistent.

